# Transcranial direct current stimulation (tDCS) in addition to walking training on walking, mobility, and reduction of falls in Parkinson’s disease: study protocol for a randomized clinical trial

**DOI:** 10.1186/s13063-021-05603-z

**Published:** 2021-09-21

**Authors:** Lucas Rodrigues Nascimento, Ester Miyuki Nakamura-Palacios, Augusto Boening, Bárbara Naeme Lima Cordeiro, Daniel Lyrio Cabral, Alessandra Swarowsky, Guilherme Peixoto Tinoco Arêas, Wellingson Silva Paiva, Fernando Zanela da Silva Arêas

**Affiliations:** 1 Center of Health Sciences, Discipline of Physical Therapy, Universidade Federal do Espírito Santo (UFES), 1468 Marechal Campos Avenue, Maruípe, Vitória, ES 29043900 Brazil; 2 NeuroGroup, Department of Physical Therapy, Universidade Federal de Minas Gerais (UFMG), Belo Horizonte, MG Brazil; 3 Laboratory of Cognitive Sciences and Neuropsychopharmacology, Department of Physiological Sciences, Universidade Federal do Espírito Santo (UFES), Vitória, ES Brazil; 4 Department of Physical Therapy, Universidade Estadual de Santa Catarina (UDESC), Florianópolis, SC Brazil; 5 Doctor of Physical Therapy Program, Advent Health University, Orlando, USA; 6 Department of Human Physiology, Universidade Federal do Amazonas (UFAM), Manaus, AM Brazil; 7 Neurosurgery Division, Department of Neurology, Clinical Hospital, Faculty of Medicine, University of São Paulo, São Paulo, Brazil

**Keywords:** Clinical trial, Parkinson, Gait, Transcranial direct current stimulation, Rehabilitation

## Abstract

**Background:**

Transcranial direct current stimulation (tDCS) has the potential to modulate cortical excitability and enhance the effects of walking training in people with Parkinson’s disease. This study will examine the efficacy of the addition of tDCS to a task-specific walking training to improve walking and mobility and to reduce falls in people with Parkinson’s disease.

**Methods:**

This is a two-arm, prospectively registered, randomized trial with concealed allocation, blinded assessors, participants and therapists, and intention-to-treat analysis. Twenty-four individuals with Parkinson’s disease, categorized as slow or intermediate walkers (walking speeds ≤ 1.0 m/s), will be recruited. The experimental group will undertake a 30-min walking training associated with tDCS, for 4 weeks. The control group will undertake the same walking training, but with sham-tDCS. The primary outcome will be comfortable walking speed. Secondary outcomes will include walking step length, walking cadence, walking confidence, mobility, freezing of gait, fear of falling, and falls. Outcomes will be collected by a researcher blinded to group allocation at baseline (week 0), after intervention (week 4), and 1 month beyond intervention (week 8).

**Discussion:**

tDCS associated with walking training may help improve walking of slow and intermediate walkers with Parkinson’s disease. If walking is enhanced, the benefits may be accompanied by better mobility and reduced fear of falling, and individuals may experience greater free-living physical activity at home and in the community.

**Trial registration:**

Brazilian Registry of Clinical Trials (ReBEC) RBR-6bvnx6. Registered on September 23, 2019

## Background

Parkinson’s disease is the most common movement disorder and represents the second most common degenerative disease of the central nervous system, resulting from the death of dopamine-producing cells in the *substantia nigra* [1]. Disabilities associated with Parkinson’s disease such as bradykinesia, impaired balance, and walking limitations can be present at initial diagnosis and progress over time [1, 2]. In individuals with Parkinson’s disease, while walking speed, step length, and mobility are typically reduced, fear of falling and the number of falls are increased [3, 4]. If walking performance is poor, community activity may be limited, and people may become housebound and isolated from the society. In addition, falls are a major determinant of poor quality of life, reduced mobility, and reduced life expectancy in people affected by Parkinson’s disease [4].

Exercise and walking training have proved to be effective for improving walking and reducing falls early in people with Parkinson’s disease [5–7]. Non-invasive brain stimulation by transcranial direct current stimulation (tDCS), which modulates cortical excitability by applying a direct current to the skull [8], could be associated with walking training and has the potential to enhance its benefits [9]. tDCS can increase the activity of the ventroposterolateral thalamic nucleus and may influence basal ganglia function. In addition, anodal tDCS over the motor cortex alter resting membrane potentials of underlying neurons leading to an increase in cortical excitability, with immediate and long-term effects that have been proposed to help improving motor skills [8, 10, 11].

A Cochrane review [8] examined the effects of tDCS in individuals with Parkinson’s disease. Meta-analyses, based on two trials, suggested that tDCS improves motor skills, measured by the Unified Parkinson’s Disease Rating Scale (UPDRS)—part III—(MD − 14%; 95% CI − 25 to − 4), but has no effect on walking speed (SMD 0.5; 95% CI − 0.2 to 1.2) compared with sham intervention. More recently, a systematic review [12] reported that tDCS provides no clinically important benefits over walking training on walking speed, step length, or cadence in people with Parkinson’s disease. Given that most trials included individuals with mild impairments or did not investigate effects on mobility and falls, the purpose of this randomized trial is to examine the effects of the addition of tDCS to walking training on walking, mobility, and falls in individuals with moderate walking limitations due to Parkinson’s disease. The specific research questions are as follows:

1. In people with Parkinson’s disease, is walking training associated with tDCS superior to walking training alone for improving walking (speed, step length, cadence, confidence), mobility, and falls?
2. Are any benefits maintained beyond the intervention period?

## Methods

### Design

**Fig. 1:**
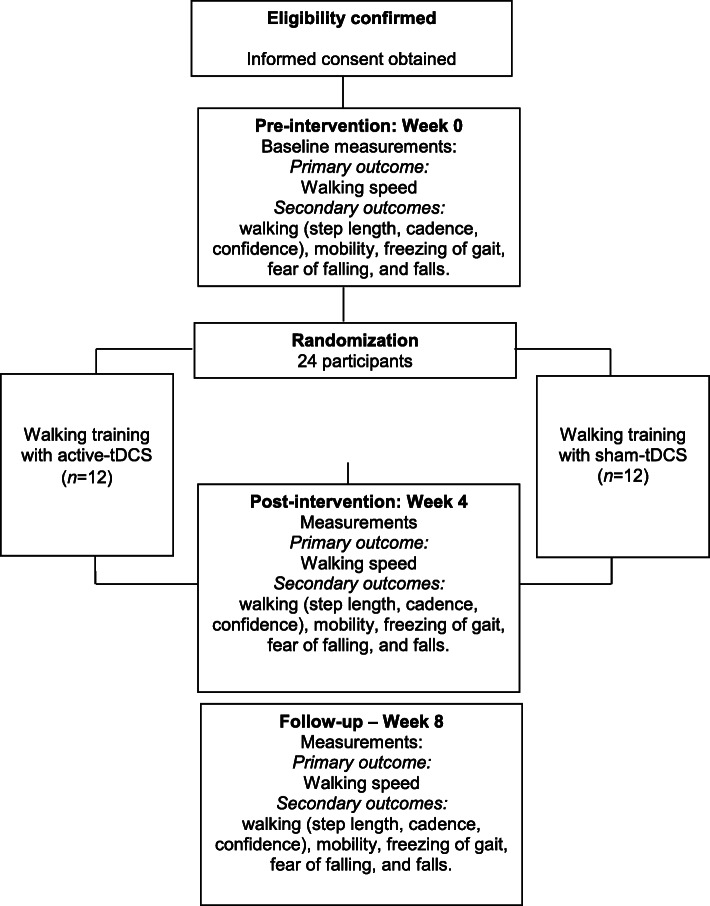
Design of the trial

A prospective, randomized controlled trial with concealed allocation, blinded assessors, participants and therapists, and intention-to-treat analysis will be carried out (Fig. 1). Community-dwelling people with Parkinson’s disease will be recruited from the general community, by means of advertisements and by screening public rehabilitation services and lists of previous observational or cross-sectional research projects. Participants will be randomly allocated into either experimental group (i.e., walking training with tDCS) or control group (i.e., walking training with sham-tDCS). Outcome measures will be collected by trained researchers at baseline (week 0), at the end of the intervention (week 4), and 1 month beyond the intervention (week 8). Measurements and interventions will be conducted during the on phase of medication. Analyses of the inclusion criteria, getting the informed consent, data collection, and statistical analyses will be carried out by researchers, who will be blind to the group allocation. All the participants will be evaluated and receive all the information regarding the interventions in a research laboratory. The study obtained ethical approval from the Institutional Research Ethical Committee (CAAE 06952819.6.0000.5060) of the Universidade Federal do Espírito Santo, Vitória, Brazil. The trial was prospectively registered at the Ensaiosclinicos.gov.br, Registry: RBR-6bvnx6 (www.ensaiosclinicos.gov.br/rg/RBR-6bvnx6/).

### Participants and therapists—inclusion and exclusion criteria

Participants will be individuals with Parkinson’s disease, who will be eligible if they:

- Are > 40 years of age
- Are able to walk at least 14 m, independently, with or without assistive devices
- Walk at speeds ≤ 1.0 m/s
- Have experienced a freezing episode over the past month, according to the part I of the New Freezing of Gait Questionnaire [13]
- Have adapted to their current anti-Parkinsonian medication for at least 2 weeks
- Provide written consent

They will be excluded if they:

- Have cognitive deficits, which will be screened by the Mini-Mental State Examination. The cutoff scores are 26 for people with high levels of education, 18 for people with elementary and middle levels, and 13 for illiterate people [14].
- Suffer from unstable cardiovascular disease or other uncontrolled chronic conditions that would interfere with the safety and conduct of the training and testing protocol or interpretation of the results.
- Had undergone deep brain stimulation.

Therapists, who will deliver the intervention, will be eligible if they have received training from the research leaders (FZSA and LRN), who have more than 10 years of clinical experience in the area of neurological rehabilitation. A research assistant will be responsible for setting the stimulator on active or sham-tDCS, so therapists delivering the intervention will be blind to the group allocation.

### Randomization

Randomization will be computer-generated, by a researcher not involved in participant recruitment, and stratified according to the baseline walking speeds: slow (≤ 0.5 m/s) and intermediate (0.51 to 1.0 m/s) walkers, to ensure an even spread between the groups (1:1 allocation). The allocation of the participants will be concealed in sequentially numbered and sealed in opaque envelopes, prepared prior to the study by a research assistant, who will not be involved in the study. After the baseline measures have been collected, participants will be randomly assigned to the experimental or control group by the treating therapist, after revealing the content of the sealed opaque envelopes. All outcomes will be measured by blinded assessors. All enrolled participants will receive a code, in order to protect confidentiality before, during, and after the trial.

### Intervention

The experimental group will undertake a task-specific walking training associated with tDCS, 30 min per day, 3 days per week, over 4 weeks, i.e., 12 sessions of tDCS. A stimulator (DC-Stimulator Plus, NeuroConn, Ilmenau, Germany, or Neuroeletrics STARTIM tCS, Barcelona, Spain) will deliver a continuous direct current by a saline-soaked pair of surface sponge electrodes (size of electrodes 35 cm^2^). The anode will be placed at the Cz position on the scalp, corresponding to the location of the supplementary motor area, in accordance with the International EEG 10/20 system [15, 16]. The cathode will be positioned over the left supraorbital area. Participants will receive electrical stimulation of 2 mA, during the walking training. The 30-min sessions of task-specific walking training will include the practicing part of the task (about 10 min), where the muscles are working in a manner similar to a full task performance and practicing the whole task (about 20 min) [17]. Table 1 shows the ten walking activities of the task-specific walking training, which will be individually tailored for each participant.

**Table 1:** Activities of the task-specific walking training

Activity	Type	Practice	Progression
Step on block	Segmented	Swing	Diminishing hand support and increasing speed, height, and/or distance
Stand on one leg then perform plantarflexion		Stance	Diminishing hand support and increasing speed
Step sideways		Lateral movement	Diminishing hand support and increasing speed and/or distance
Step sideways on a block		Lateral movement	Diminishing hand support and increasing speed, height, and/or distance
Walk on footprints	Complete	Whole task	Increasing step length
Walk with an auditory cue		Whole task	Increasing cadence
Walk and turn		Whole task	Increasing speed
Walk and cross obstacles		Whole task	Increasing speed
Dual-task walk		Whole task	Increasing speed and cognitive/motor demands
Overground and treadmill speed walk		Whole task	Increasing speed

The control group will undertake the task-specific walking training associated with a sham-tDCS. The control group will receive the same walking training, electrode positioning, and testing schedule as the experimental group. This will avoid bias related to the type and amount of attention given to the participants. If the addition of tDCS proves to be effective, the control group may receive the experimental training program after the experiment is complete.

The intervention will be undertaken in Clinics of Physiotherapy at the Universidade Federal do Espírito Santo. To encourage the participants to comply with the protocol, both groups will be asked to sign a symbolic contract of commitment to the proposed protocol. Participants will not be informed whether they are receiving tDCS or sham-tDCS.

### Primary outcome

The primary outcome is comfortable walking speed, measured by the 10-m Walk Test, and reported in m/s. The participants will be instructed to walk at their “comfortable speed” along a 14-m hallway, and the time to cover the central 10 m will be recorded with a digital stopwatch and converted to speed [18]. After a practice trial, the value obtained during a single test will be used for analysis [19].

### Secondary outcomes

Secondary outcomes are walking step length, walking cadence, walking confidence, mobility, freezing of gait, fear of falling, and falls.

Walking step length and cadence will be measured using the 10-m Walk Test. Step length will be calculated by dividing the covered distance, i.e., 10 m, by the number of steps to cover the distance, and reported in meters. Walking cadence will be calculated by dividing the number of steps by the time to cover the distance, i.e., 10 m, and reported in steps/min.

Walking confidence will be measured using the Brazilian version of the modified Gait Efficacy Scale and reported as scores ranging from 10 to 100. This scale is a 10-item measure that addresses the perception of the level of confidence in walking during challenging circumstances. The items include walking on a level surface and on grass, stepping over an obstacle, stepping up and down a curb, ascending and descending stairs (with and without a handrail), and walking over a long distance. The items are individually scored on a 10-point Likert scale, with 1 indicating “no confidence” and 10 indicating “complete confidence,” [20, 21].

Mobility will be measured by the Timed-up and Go Test (TUG) and reported as seconds. The participants will be seated in a chair with their backs against the chair back. On the command “go,” the participants will be instructed to rise from the chair, walk 3 m at a comfortable and safe pace, turn, walk back to the chair, and sit down. After a practice trial, the value obtained during a single test will be used for analysis [19, 22].

Freezing of gait will be measured using parts II and III of the New Freezing of Gait Questionnaire and reported as scores ranging from 0 to 28, where higher scores indicate worse episodes of freezing. The New Freezing of Gait Questionnaire is a reliable tool to detect and evaluate the impact and severity of freezing of gait, when applied to patients (ICC = 0.88) or caregivers (ICC = 0.97) [13].

Fear of falling will be measured using the Brazilian version of the Falls Efficacy Scale – International (FES-I Brazil) and reported as scores ranging from 16 to 64, where higher scores indicate greater fear of falling. The FES-I Brazil measures the fear of falling during 16 activities of daily living and has appropriate internal consistency (*α* = 0.93) and intra- and inter-examiner reliability (ICC = 0.84 and ICC = 0.91) [23].

The number of falls will be recorded by the use of a “falls diary” [24], and the proportion of fallers in each group will also be compared. All participants will receive weekly calendars on entry to the study, with instructions to record the following events: number of falls, visits by or to nursing and allied health personnel, and hospitalizations. Participants will be asked to return the completed calendar weekly to a researcher unaware of the group allocation.

### Data monitoring body

An independent researcher, who will be blind to the group allocations, will monitor any adverse effects and perform database management and statistical analyses. The treating therapists will be responsible for the monitoring of doses and compliance.

### Sample size estimation

Twenty-four participants will be recruited, with walking speed as the primary outcome. The sample size has been calculated, to reliably detect a between-group difference of 0.18 m/s in walking speed, with 80% power, at a two-tailed significance level of 0.05. In previous trials [9, 16, 25] with similar samples of community-dwelling people with Parkinson’s disease who received tDCS associated with walking training, the mean walking speed of the participants was 0.73 m/s (SD 0.15 m/s), measured by a timed-walking measure. The least number of participants needed to detect a 0.18 m/s difference between two independent groups, which would indicate a change in the UPDRS walking category [26], is 11 per group, i.e., 22 participants in total. Based on the assumption that about 10% of participants may drop out during the study, a target of 24 participants in total has been set.

### Statistical analyses

Data collection will yield eight variables: walking speed (m/s), walking step length (m), walking cadence (steps/min), walking confidence (modified Gait Efficacy Scale score; 10–100), mobility (TUG; seconds), freezing of gait (New Freezing of Gait Questionnaire; 0–28), fear of falling (FES-I Brazil score; 16–64), and number of falls. There are two factors (group × time), with repeated measures on the time factor. Two-way analyses of variance with repeated measures at all time points for all outcomes will be reported to evaluate the statistical significance of the between-group differences. The mean between-group differences, along with 95% confidence intervals, will be reported for all outcomes. The effect of the intervention will be calculated based on intention-to-treat analyses.

### Study organization and funding

This trial will be conducted according to relevant ethical frameworks and has received approval from the institutional ethical review board. It is funded by the Brazilian National Funding Agency: Fundação de Amparo à Pesquisa e Inovação do Espírito Santo (FAPES). The results will be submitted for publication in journals related to the area of neurorehabilitation, and access to the final trial dataset may be obtained from the authors based upon reasonable request.

## Discussion

This trial will examine the efficacy of the addition of tDCS to walking training for improving walking, mobility, and falls in people with Parkinson’s disease. Although previous studies [9, 10, 15, 16, 27] have investigated the combined effect of tDCS and walking training, methodological shortcomings (e.g., design and very small sample sizes), characteristics of interventions (e.g., duration of intervention, type of exercises), and characteristics of the participants (e.g., level of disabilities) prevent drawing clear conclusions, which could help clinicians in their decision-making process. In addition, many trials did not investigate whether benefits carry over to improving mobility and falls. In response to this challenge, a triple-blinded randomized trial will be conducted. High internal validity is expected, due to randomization, concealed allocation, blinding of assessors, participants and therapists, intention-to-treat analysis [28], and appropriate sample size.

Previous studies suggest that tDCS modulates cortical excitability during stimulation by non-synaptic changes of the cells, and increasing evidence indicates that the aftereffects of tDCS are driven by synaptic modification [29]. Synaptic plasticity could, therefore, induce long-lasting excitability changes in the central nervous system. Although the neurophysiological effects of tDCS have been identified and its safety has been proved [29, 30], the clinical effects remain unclear. This trial focuses on identifying the effects of the addition of tDCS on walking parameters commonly affected in individuals with Parkinson’s disease [3, 31]. If neurophysiological benefits of tDCS are carried over to clinical benefits, clinicians will assure an important tool to help reduce disabilities related to the continuous death of dopamine-producing cells in the *substantia nigra*. Less expensive portable devices are currently available, making direct current potentially ideal as an adjunct to other physical interventions [30].

This trial has some limitations. The experimental and control interventions consist of walking exercises delivered three times per week over 4 weeks and, therefore, depend on participants’ motivation, adherence, and commitment. Strategies to encourage participants to comply with the protocol, such as contracts and phone calls, are planned.

In conclusion, the results of this trial may result in an important advance in neurological rehabilitation. First, an adjunct intervention may help improve walking of slow and intermediate walkers with Parkinson’s disease. Second, if walking is enhanced, the benefits may be accompanied by better mobility and reduced fear of falling. Individuals may experience greater free-living physical activity at home and in the community, increased social interactions, and increased ability to engage in work and leisure activities [32, 33], which is the ultimate goal for both patients and rehabilitation professionals.

## Trial status

Recruitment has started in September 2019 according to the registry RBR-6bvnx6, version 1.0, September 23, 2019. At the time of manuscript submission, the expected duration of the study, including enrollment and statistical analysis, should be 5 years. The approximate date of planned recruitment completion is December 31, 2023.

## Data Availability

The study investigators have full access to the study datasets. The datasets used and analyzed during the study are available from the corresponding author on reasonable request; however, any information shared will be blinded to any identifying participant information. The trial results will be communicated to healthcare professionals and other relevant groups via publications, reporting in results databases, and presenting the data during the medical congresses and conferences.

## Abbreviations

tDCS: Transcranial direct current stimulation
UPDRS: Unified Parkinson’s Disease Rating Scale
TUG: Timed-up and Go Test
FES-I: Falls Efficacy Scale – International
steps/min: Steps per minute
