# Correction to: Transcranial direct current stimulation (tDCS) in addition to walking training on walking, mobility, and reduction of falls in Parkinson’s disease: study protocol for a randomized clinical trial

**DOI:** 10.1186/s13063-021-05720-9

**Published:** 2021-10-18

**Authors:** Lucas Rodrigues Nascimento, Ester Miyuki Nakamura-Palacios, Augusto Boening, Bárbara Naeme Lima Cordeiro, Daniel Lyrio Cabral, Alessandra Swarowsky, Guilherme Peixoto Tinoco Arêas, Wellingson Silva Paiva, Fernando Zanela da Silva Arêas

**Affiliations:** 1 Center of Health Sciences, Discipline of Physical Therapy, Universidade Federal do Espírito Santo (UFES), 1468 Marechal Campos Avenue, Maruípe, Vitória, ES 29043900 Brazil; 2 NeuroGroup, Department of Physical Therapy, Universidade Federal de Minas Gerais (UFMG), Belo Horizonte, MG Brazil; 3 Laboratory of Cognitive Sciences and Neuropsychopharmacology, Department of Physiological Sciences, Universidade Federal do Espírito Santo (UFES), Vitória, ES Brazil; 4 Department of Physical Therapy, Universidade Estadual de Santa Catarina (UDESC), Florianópolis, SC Brazil; 5 Doctor of Physical Therapy Program, Advent Health University, Orlando, USA; 6 Department of Human Physiology, Universidade Federal do Amazonas (UFAM), Manaus, AM Brazil; 7 Neurosurgery Division, Department of Neurology, Clinical Hospital, Faculty of Medicine, University of São Paulo, São Paulo, Brazil

**Correction to: Trials 22, 647 (2021)**

**https://doi.org/10.1186/s13063-021-05603-z**

Following the publication of the original article [1], we were notified that Bárbara Naeme Lima Cordeiro was missed from being mentioned on the 4th place in the authorship list.

Bárbara Naeme Lima Cordeiro has participated in the writing of the design of the protocol.

The original article has been corrected.
